# Ambient sulfur dioxide and daily outpatient visits for allergic conjunctivitis: a multi-city time-stratified case-crossover study in China

**DOI:** 10.1186/s12889-025-25700-x

**Published:** 2025-11-22

**Authors:** Yun Qiu, Zuqiong Song, Liujie Zhu, Zhen Wang, Wei Shan, Yanfeng Liao, Tao Zhang, Wenhui Liu, Hui Guo, Zhen Ding, Zengliang Ruan

**Affiliations:** 1 Department of Public Health, School of Medicine, Nanjing University of Chinese Medicine, Nanjing, Jiangsu China; 2 State Key Laboratory of Reproductive Medicine and Offspring Health, Nanjing Medical University, Nanjing, Jiangsu China; 3 Huizhou first Maternal and Child Health Care Hospital, Huizhou, Guangdong China; 4 Jiangsu Provincial Key Laboratory of Critical Care Medicine, Southeast University, Nanjing, Jiangsu China; 5 Key Laboratory of Environmental Medicine and Engineering of Ministry of Education, School of Public Health, Southeast University, Nanjing, Jiangsu China; 6 Department of Ophthalmology, Suzhou Municipal Hospital & The Affiliated Suzhou Hospital of Nanjing Medical University, Nanjing Medical University, Suzhou, Jiangsu China; 7 Administrative Office, Suzhou Municipal Hospital & The Affiliated Suzhou Hospital of Nanjing Medical University, Nanjing Medical University, Suzhou, Jiangsu China; 8 Guangzhou Yuexiu District Children’s Hospital, Guangzhou, Guangdong China; 9 Department of Ophthalmology, Jiangnan University Medical Center, Wuxi, Jiangsu China; 10 Department of Ophthalmology, Qilu Hospital of Shandong University, Jinan, Shandong China; 11 Jiangsu Provincial Center for Disease Control and Prevention, 172 Jiangsu Rd, Nanjing, 210009 Jiangsu China; 12 Department of Medical Epidemiology and Biostatistics, Karolinska Institutet, Stockholm, Sweden; 13 Department of Epidemiology, School of Public Health, Sun Yat-Sen University, Guangzhou, Guangdong China

**Keywords:** Sulfur dioxide, Allergic conjunctivitis, Ocular surface, Outpatient visit, Case-crossover

## Abstract

**Background:**

Evidence remains sparse and inconclusive regarding the acute effect of ambient sulfur dioxide (SO_2_) exposure on allergic conjunctivitis (AC). The main objective of this study was to assess the temporal relationship between acute SO_2_ exposure and AC risk.

**Methods:**

This study employed a case-crossover design incorporating daily data on outpatient visits for AC from five hospitals across five Chinese cities, spanning from January 2014 to December 2022. Daily pollution and meteorological data were retrieved through national air quality surveillance system. To examine the link between SO_2_ and AC, we utilized conditional logistic regression models combined with random-effects meta-analyses.

**Results:**

Over the study period, there were 109,985 outpatient visits for AC, with 63,423 (57.7%) males and 46,562 (42.3%) females. We observed a significant positive association between SO_2_ and AC outpatient visits, with an adjusted odds ratio (OR) of 1.045 (95% CI: 1.011, 1.079) for every standard deviation (SD) increase in SO_2_ concentrations. This positive association remained consistent across sex, age, and season. Furthermore, the association remained significant and robust when adjusting for other pollutants, such as fine particulate matter, carbon monoxide, nitrogen dioxide and ozone, in both two-pollutant and multi-pollutant models.

**Conclusions:**

This is the largest study in China to demonstrate that short-term SO_2_ exposure increases the risk of AC. Our findings emphasize that reducing SO_2_ pollution is important to protect ocular health and provide insights for future standards and policies.

**Supplementary Information:**

The online version contains supplementary material available at 10.1186/s12889-025-25700-x.

## Introduction

Allergic conjunctivitis (AC) is an inflammatory condition in the surface of the eye. It is estimated to affect 30% of the general population and between 6% and 40% of children, with an expected annual increase [1, 2]. Symptoms of AC include redness, itching, sensitivity to light, and blurred vision. It often occurs alongside other allergic conditions such as allergic rhinitis, asthma, and atopic dermatitis [3, 4]. AC can negatively impact the quality of life of patients [5], affect sleep quality [6], and lead to decreased educational performance and economic productivity, ultimately resulting in significant socioeconomic costs [1].

Air pollution is a major global health threat, causing 6.67 million deaths worldwide in 2019 [7]. China faces unprecedented challenges due to increasing air pollution, particularly with the rapid development of urbanization and industrialization [8]. Ambient sulfur dioxide (SO_2_) pollution has consequently become an environmental concern in China. SO_2_ is a colorless, volatile gaseous pollutant with a sharp, pungent odor. Major anthropogenic emission sources include coal and petroleum combustion in power plants and industrial facilities, while natural emissions predominantly result from volcanic activity and biomass burning [9]. There is growing concern about the harmful effects of SO_2_ on the conjunctiva, as it is constantly exposed to the atmospheric environment, potentially causing damage. For example, ambient SO_2_ exposure has been linked to an elevated risk of conjunctivitis in population-based studies [10, 11], with experimental evidence indicating that the underlying mechanism may involve oxidative stress and lipid peroxidation [12]. Therefore, it is crucial and urgent to control and intervene in SO_2_ pollution to alleviate its burden on health.

The relationship between short-term exposure to ambient SO_2_ and AC has been understudied and remains unclear. A study in Northeast China found a positive correlation between SO_2_ exposure and AC, suggesting that SO_2_ poses a significant environmental risk in China [13]. However, this study identified AC cases based on drug sales rather than direct diagnoses. Another retrospective study in Shanghai found no relation between SO_2_ and AC visits [14]. Additionally, a case-crossover study from Israel demonstrated that SO_2_ exposure appeared likely to be related with vernal keratoconjunctivitis [15]. The existing literature on the relationship between SO_2_ exposure and AC among Chinese populations has been constrained by several limitations such as imprecise or inconsistent diagnostic criteria, a narrow geographical scope, limited sample sizes, and heterogeneous demographic characteristics. Therefore, further research is needed to confirm the relationship between SO_2_ exposure and AC in Chinese cities, and our studies are designed to overcome these limitations by employing participants diagnosed by clinical doctor, covering a wider geographical area, including a larger sample, and ensuring homogeneous demographic characteristics. This will provide unique insights and significantly advances for the current knowledge in this field.

This study employed a case-crossover design incorporating data on outpatient visits for AC from five cities in China between 2014 and 2022. We aimed to investigate whether short-term exposure to SO_2_ is linked to an elevated risk of AC among Chinese residents.

## Methods

### Study design

We utilized a case-crossover design to examine the correlation between daily SO_2_ exposure and outpatient visits for AC. This design has been commonly used to assess the acute effects of environmental pollutants on human health [16–18]. In this approach, each patient’s date of outpatient visit served as the “case day,” while control days were defined as other weekdays within the same calendar month. For example, if a patient sought care for AC on Tuesday, June 9, 2020, all other Tuesdays in June 2020 (June 2, 16, 23, and 30) were designated as control days. This design inherently controls for individual-level factors (e.g., sex, age, lifestyle, and socioeconomic status) that remain stable over short periods and further adjusts for potential confounders such as seasonal patterns, long-term trends, and day-of-week effects [19, 20].

### Outpatient data

**Fig. 1:**
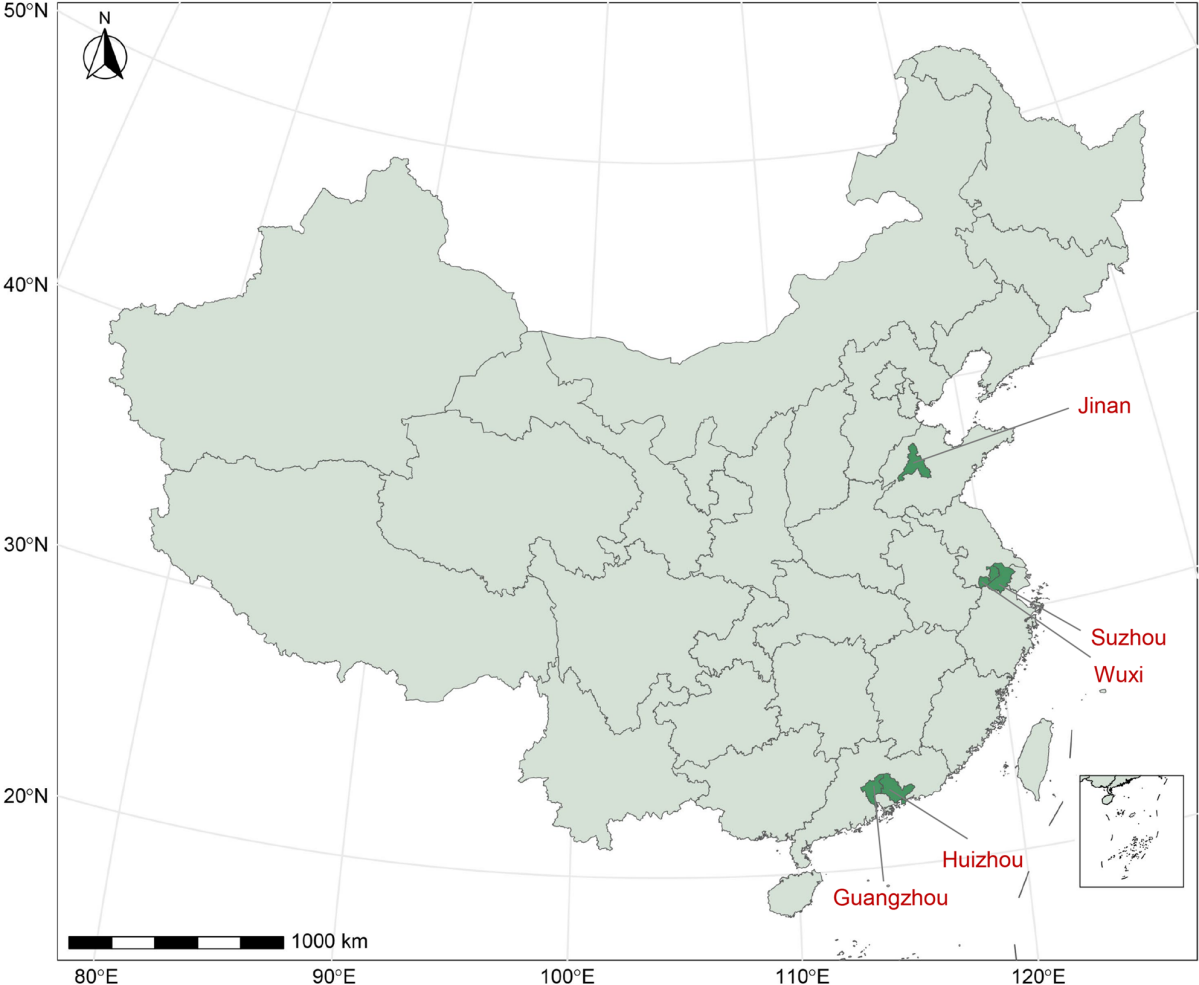
Geographical locations of five cities

Outpatient visit data were collected from electronic medical records of five hospitals across five Chinese cities including Suzhou, Huizhou, Guangzhou, Jinan and Wuxi, along with patient sex, age, and date of visit. The geographical distribution of the included cities is presented in Fig. 1, while a more detailed map illustrating the specific locations of air pollution monitoring stations within these cities is provided in Figure S1. These cities were selected based on the accessibility of comprehensive datasets encompassing air pollution metrics, meteorological parameters, and AC-related outpatient records. They are distributed across southeastern coastal China, exhibiting similar geographic and climatic patterns [21, 22]. Among the studied cities, Guangzhou, Huizhou, Suzhou, and Wuxi exhibit typical subtropical monsoon climate characteristics, marked by hot-humid summers and mild-dry winters. In contrast, Jinan demonstrates a temperate monsoon climate pattern, characterized by four distinct seasons with hot summers, cold winters, and moderate precipitation levels [23, 24]. The diagnosis of AC cases was carried out by experienced physicians in the ophthalmology outpatient departments of the included hospitals. These physicians made their diagnoses based on a thorough assessment that encompassed clinical signs and symptoms, detailed medical history, and physical examination findings. When necessary, they also utilized other relevant ancillary tests to support their diagnosis. The identification of AC cases from the outpatient records were conducted using both the Chinese translation of the terminology “allergic conjunctivitis” and the specific ICD-10 code H10.1. The study protocol was approved by the Ethics Committee in School of Public Health of Sun Yat-Sen University (No.: 2019 − 149). Individual consent was waived because all of the analyses were based on deidentified retrospective hospital outpatient records.

### Exposure assessment

Hourly SO_2_ concentrations data were sourced from China’s national air quality monitoring platform, from which daily average concentrations were calculated for each city. This platform provides real-time pollutant data across all national monitoring sites in China. In the absence of specific residential addresses, exposure concentrations were assigned to cases based on the city-wide average of air pollutant concentrations from all available monitoring stations in their city of residence. To account co-exposure to other pollutants, we also extracted daily averages of carbon monoxide (CO), nitrogen dioxide (NO_2_), and fine particulate matter (PM_2.5_), as well as maximum 8-h mean concentrations of ozone (O_3_). To ensure data completeness and reliability, we required a minimum of 20 valid hourly records per day for calculating daily average concentrations, in accordance with the principles outlined in GB 3095 − 2012. Data on temperature and humidity were obtained from the Chinese weather data sharing platform.

### Statistical analysis

Descriptive statistics summarized AC patients by sex, age and season (using counts and proportions), while continuous variable such as the concentrations of pollutants (SO_2_, CO, NO_2_, O_3_, PM_2.5_) and meteorological conditions (temperature, relative humidity) were described by means, standard deviation (SD), quartiles, and ranges. Spearman’s correlation coefficients were calculated to assess relationships between air pollutants and meteorological factors.

The primary analysis used a two-stage approach to estimate associations between short-term SO_2_ exposure and AC outpatient visits. First, city-specific conditional logistic regression models were fitted, adjusting for long-term trends, public holidays, day-of-the-week effects, and spline functions for temperature with six degrees of freedom and humidity with three degrees of freedom, according to previous experiences from similar studies [25, 26]. Our prior results have suggested that changes in degrees of freedom have a minimal impact on OR estimates [27]. Second, the overall effect was averaged using random-effects meta-analysis, a method widely employed in multi-city studies to account for both within- and between-city variability. Pooled odds ratios (OR) with 95% confidence interval (CI) per SD increase in SO_2_ concentrations were reported. Exposure-response curves were generated for all cities combined and individually through conditional logistic regression controlling for confounding factors and incorporating natural spline smoothing functions for SO_2_ with a degree of freedom of three. Missing rates of variables in this study were less than 0.45%, including O_3_ (0.45%), CO (0.03%), and temperature (0.43%), deemed acceptable based on prior studies [28, 29]. In our subsequent data analyses, we opted to exclude cases with missing data to ensure the robustness and integrity of our results.

Subgroup analyses stratified by sex (male and female), age (< 6, 6–17, 18–59, ≥60 years) and season (spring, summer, autumn, and winter) were conducted. The consistency of effect estimates among subgroups was assessed by heterogeneity test with Cochran’s Q tests and I^2^ statistics, in accordance with established practices [30]. In addition, we performed several sensitivity analyses to validate our main results. First, we evaluated that lagged SO₂ exposures (same-day [Lag0] to 14 days prior [Lag14], and moving averages [Lag0-1 to Lag 0-14 ]); Second, two-pollutant models adjusting for PM_2.5_, CO, NO₂, or O₃ were fitted individually; Additionally, we conducted a multi-pollutant model by simultaneously incorporating all four pollutants.

All analyses were performed in R (v4.3.1) with two-sided hypothesis testing and a significance standard of *P* < 0.05.

## Results

This study analyzed 109,985 outpatient visits for AC across five Chinese cities, including 63,423 (57.7%) males and 46,562 (42.3%) females, with the largest age group under 6 years (46,154 cases, 42.0%). Seasonal distribution showed peaks in autumn (30.1%) and summer (28.3%), followed by spring (25.8%) and winter (15.9%) (Table 1). The total number of AC case across five cities was 59,095 in Guangzhou, 2,424 in Huizhou, 16,328 in Suzhou, 26,405 in Wuxi, and 5,733 in Jinan.

**Table 1:** Characteristics of participants in this study

Characteristics	Total
Sex	
Male	63,423 (57.7)
Female	46,562 (42.3)
Age, years	
< 6	46,154 (42.0)
≥6 & <18	36,087 (32.8)
≥18 & <60	22,721 (20.7)
≥60	5,022 (4.6)
Season	
Spring	28,338 (25.8)
Summer	31,117 (28.3)
Autumn	33,051 (30.1)
Winter	17,479 (15.9)
Total	109,985 (100.0)

As shown in Figure S2-S6A, the AC outpatient visits increased annually in most cities during the study period, with minimal seasonal variation. In addition, air pollutant concentrations—including SO₂, CO, NO₂, PM_2.5_, and O₃—generally decreased over the study period but displayed distinct seasonal fluctuations (Figures S2-S6B). Specifically, daily mean SO_2_ levels averaged 12.2 ± 9.8 (range: 2.8 to 59.4) µg/m^3^ across cities, with Jinan (25.7 ± 13.6 µg/m^3^) and Huizhou (6.7 ± 1.4 µg/m^3^) representing the highest and lowest levels. Meteorological conditions during the study period remained relatively stable, with average temperatures of 19.5 ± 8.6 ℃ and humidity of 71.9 ± 16.0% (Table 2 and S1).

**Table 2:** Summary statistics of daily air pollution and weather conditions

Variables	Mean ± SD	Minimum	First quartile	Median	Third quartile	Maximum
SO_2_ (µg/m^3^)	12.2 ± 9.8	2.8	6.4	8.5	13.9	59.4
CO (mg/m^3^)	0.8 ± 0.3	0.2	0.6	0.7	0.9	5.4
NO_2_ (µg/m^3^)	37.2 ± 18.4	3.2	23.0	34.6	48.0	135.2
O_3_ (µg/m^3^)	94.7 ± 47.0	2.9	58.6	88.6	125.9	274.0
PM_2.5_ (µg/m^3^)	38.5 ± 27.4	2.0	20.3	31.0	48.5	276.8
Temperature (℃)	19.5 ± 8.6	−13.2	13.6	21.3	26.6	35.3
Relative humidity (%)	71.9 ± 16.0	14.3	63.6	74.3	83.5	100.0

Correlation analysis identified strong positive relationships between SO_2_ and primary pollutants (CO, NO_2_, PM_2.5_; Spearman’s r range: 0.56 to 0.64), weak associations with O_3_ (*r* = 0.11), and inverse correlations with temperature (*r* = −0.20) and humidity (*r* = −0.39). Air pollutants CO, NO_2_ and PM_2.5_ showed moderate-to-high intercorrelation (r range: 0.63 to 0.73), all negatively associated with meteorological factors (r range: −0.03 to −0.41). In addition, O_3_ exhibited weak-to-moderate linkages with other pollutants and meteorological factors (Figure S7).

**Fig. 2:**
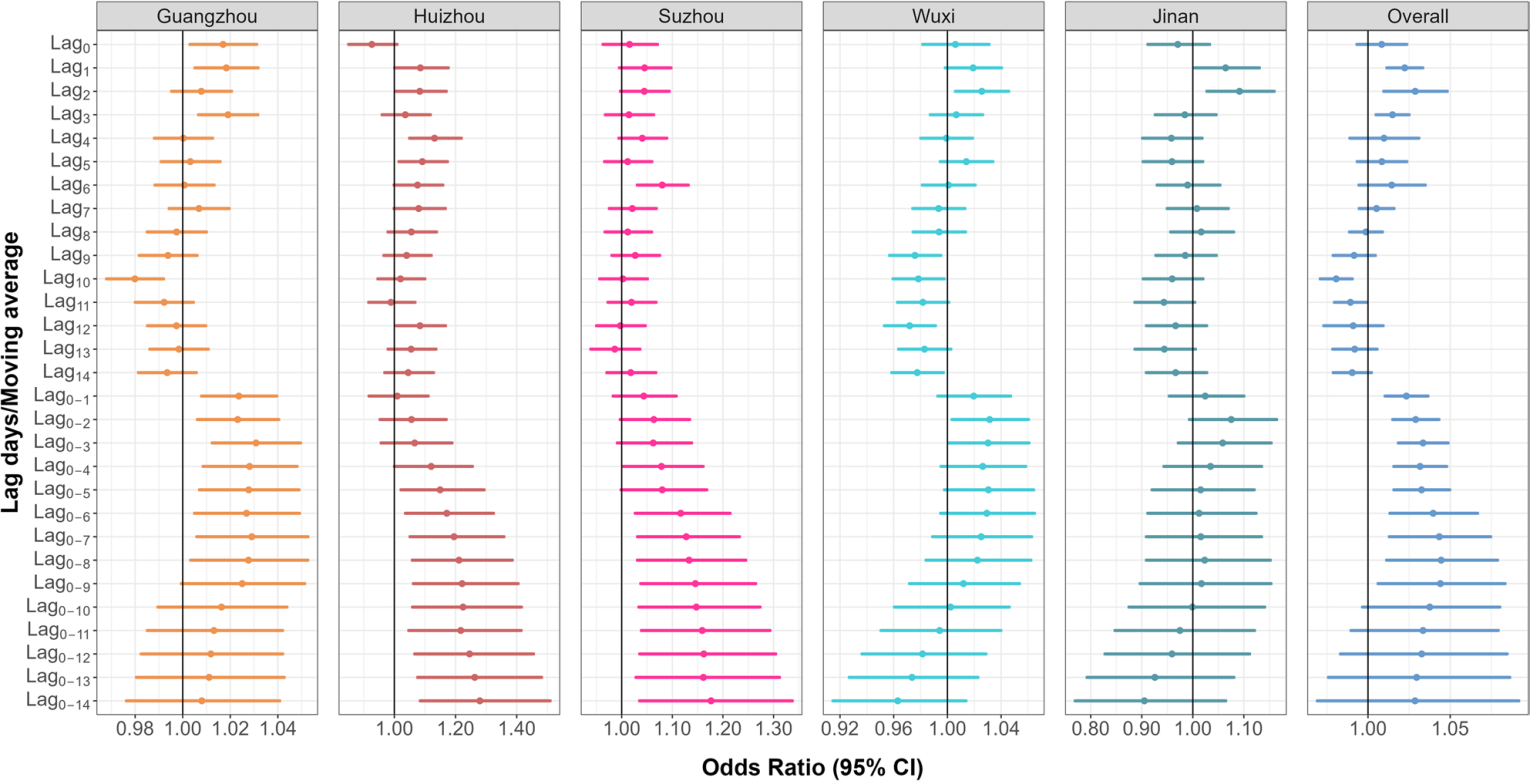
Association between sulfur dioxide exposure and allergic conjunctivitis outpatient visits. The results are shown as odds ratios per standard deviation increase

**Fig. 3:**
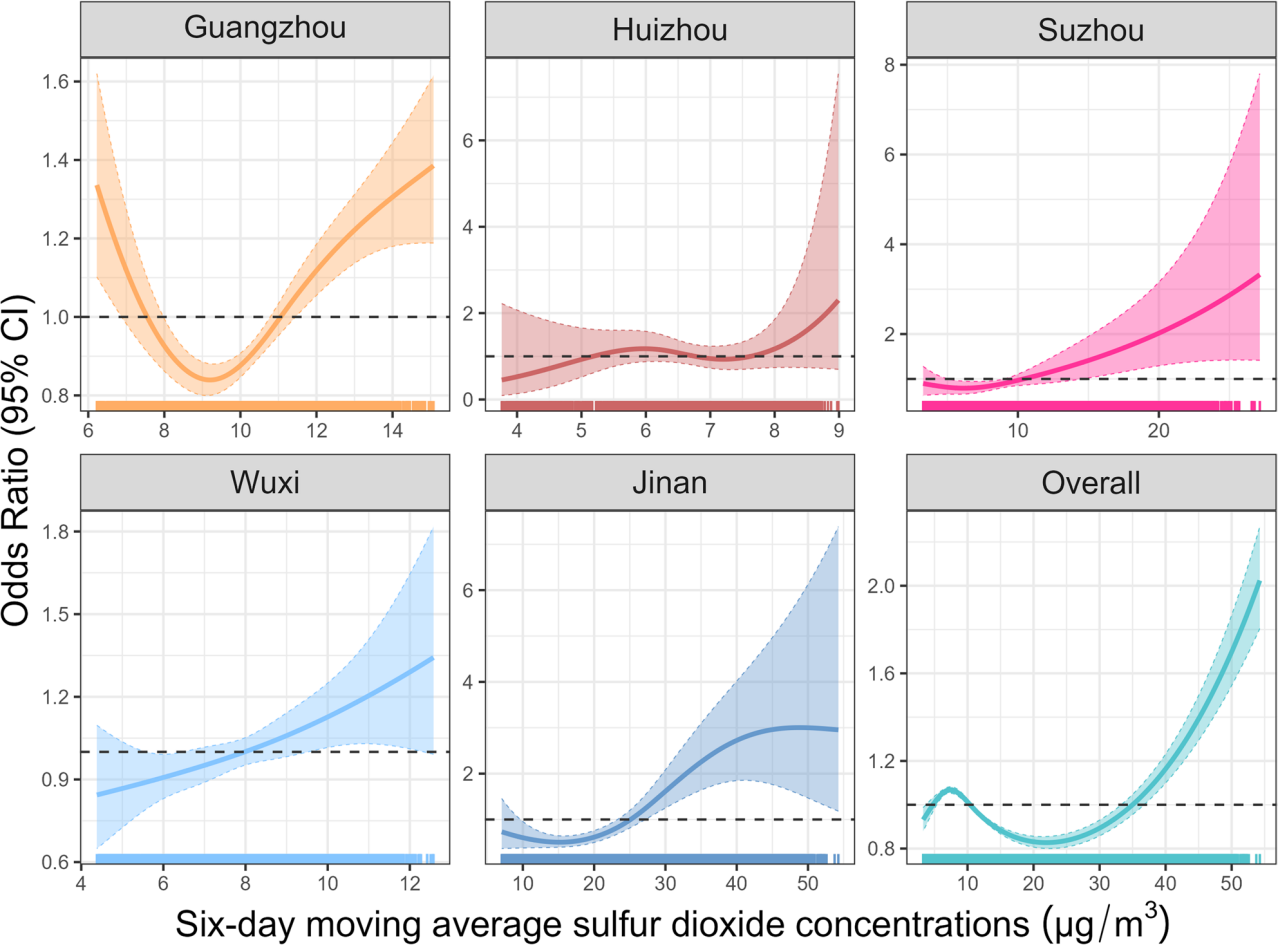
Exposure-response relationship between sulfur dioxide exposure and allergic conjunctivitis. The line represents the point estimates of odds ratio, and shading areas indicates corresponding 95% CIs

Our main analyses indicated positive association between daily mean SO₂ levels and AC outpatient visits, with the 8-day moving average (lag_0 − 8_) showing the strongest effect (Fig. 2), thus lag_0 − 8_ results were reported in the subsequent analyses. A 1-SD increase in SO₂ exposure was linked to a 4.5% increment in AC outpatient risk (OR = 1.045, 95% CI: 1.011–1.079) in pooled models (Fig. 2 and Table S2). The exposure-response curves for the association between SO₂ and AC risk presented in Fig. 3 demonstrate a generally consistent, approximately linear dose-dependent relationship across most cities and in the overall pooled analysis, where the risk of AC appears to increase steadily with rising SO₂ concentrations. Notably, in Guangzhou, the odds ratio initially decreases to a minimum around 8–9 µg/m³ before increasing, suggesting a potential threshold effect. In Suzhou, Wuxi and Jinan, the curves show a more gradual, near-linear increase in AC risk, highlighting a pronounced dose-response relationship without clear thresholds. The overall panel combines these city-specific trends, showing a general upward trend with increasing SO₂ levels, though the magnitude and shape of the response vary by location.

**Fig. 4:**
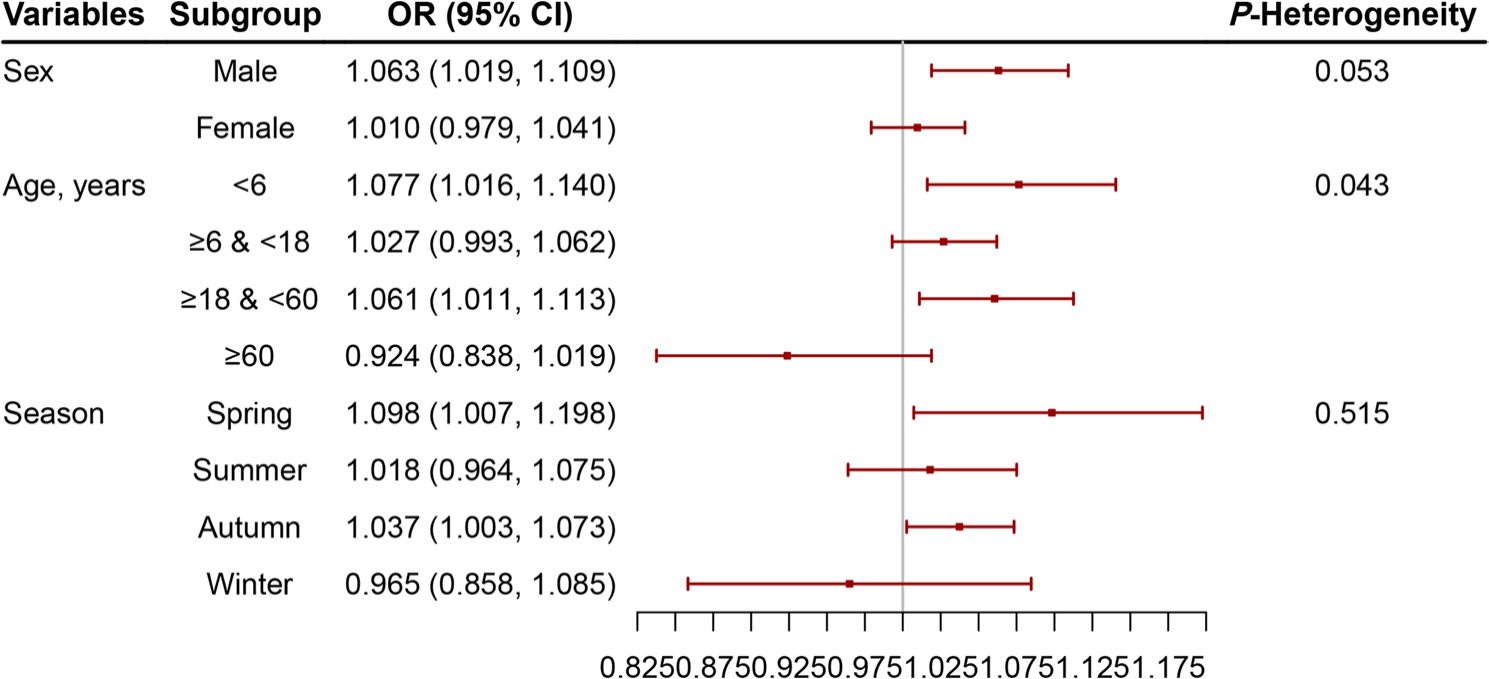
Stratified analysis of sulfur dioxide exposure effects on allergic conjunctivitis by sex, age, and season. The results are shown as odds ratios per standard deviation increase

The results of the stratified analyses were presented in Fig. 4 and Table S3, indicating increased vulnerabilities in specific patient subgroups and seasons. Specifically, significant positive associations between SO_2_ exposure and AC were found in males (OR = 1.063, 95% CI: 1.019–1.109), children under 6 years of age (OR = 1.077, 95% CI: 1.016–1.140), adults aged 18–60 (OR = 1.061, 95% CI: 1.011–1.113), and during spring (OR = 1.098, 95% CI: 1.007–1.198) or autumn (OR = 1.037, 95% CI: 1.003–1.073). We observed a notable heterogeneity among age groups (*P* = 0.043). However, no significant heterogeneity between subgroups were detected in sex (*P* = 0.053), and season groups (*P* = 0.515). Sensitivity analyses demonstrated the robustness of our main findings through additionally adjusting for PM_2.5_, CO, NO_2_ and O_3_ in the two-pollutant model. Additionally, inclusion of all four pollutants did not alter the association (OR = 1.057, 95% CI: 1.018–1.098) (Table 3).

**Table 3:** Associations between sulfur dioxide and allergic conjunctivitis in the single-pollutant, two-pollutant and multipollutant models

	Guangzhou	Huizhou	Suzhou	Wuxi	Jinan	Overall
	OR (95% CI)	OR (95% CI)	OR (95% CI)	OR (95% CI)	OR (95% CI)	OR (95% CI)
Single-pollutant model	1.028 (1.003, 1.053)	1.211 (1.059, 1.385)	1.133 (1.031, 1.245)	1.022 (0.984, 1.062)	1.023 (0.909, 1.152)	1.045 (1.011, 1.079)
Two-pollutant model						
+PM_2.5_	1.015 (0.991, 1.041)	1.219 (1.064, 1.397)	1.132 (1.030, 1.244)	1.038 (0.999, 1.079)	1.087 (0.960, 1.231)	1.053 (1.015, 1.093)
+CO	1.025 (1.001, 1.050)	1.210 (1.058, 1.384)	1.135 (1.033, 1.247)	1.026 (0.987, 1.065)	1.056 (0.935, 1.194)	1.047 (1.013, 1.082)
+NO_2_	1.019 (0.994, 1.044)	1.229 (1.074, 1.407)	1.144 (1.040, 1.258)	1.040 (1.000, 1.082)	1.028 (0.911, 1.160)	1.054 (1.015, 1.094)
+O_3_	1.027 (1.002, 1.052)	1.211 (1.057, 1.387)	1.134 (1.032, 1.247)	1.027 (0.988, 1.067)	1.008 (0.895, 1.135)	1.045 (1.011, 1.079)
Multipollutant model	1.017 (0.992, 1.042)	1.229 (1.072, 1.410)	1.138 (1.034, 1.252)	1.044 (1.003, 1.087)	1.073 (0.947, 1.215)	1.057 (1.018, 1.098)

## Discussion

This study identified a significant relationship between short-term SO_2_ exposure and increased risk of AC, with a dose-dependent relationship observed. These effects remained consistent across sex, age, and seasonal subgroups. Notably, the association persisted after adjustment for major co-pollutants, demonstrating its robustness. This is the largest multi-city study in China to analyze AC visits associated with short-term SO_2_ exposure.

Previous research has yielded mixed findings regarding SO₂ exposure and conjunctivitis risk. For example, several studies have reported that short-term exposure to ambient SO_2_ was positively associated with the risk of conjunctivitis in the general population [10, 11, 31], which aligns with our findings. However, in 2019, a meta-analysis of 12 studies found no correlation between SO_2_ exposure and conjunctivitis risk [32]. Similarly, a recent time-series study detected no association in both single and multi-pollutant models [33]. As a most common conjunctivitis subtype, AC differs in prevalence patterns and mechanisms from other forms [34]. Limited studies have explored SO_2_ exposure and the outpatient visits for AC specifically. Our findings align with a prior study in Northeast China reporting a strong positive correlation between SO_2_ and the occurrence of AC, which also found that SO_2_ threshold was lower than the current air quality standard in China [13]. However, this study lacked adjustment for confounding factors, and the identification of AC cases relied on sales data of the antiallergic medications in the treatment of AC, which was an indirect method and lacked of detailed individual’s information. Conversely, other research found no elevated AC risk associated with SO_2_ exposure in general or pediatric populations [14, 35]. The discrepancies between these studies and ours may stem from several factors, including heterogeneity in the study area and sample size, varied population characteristics, different diagnostic criteria and air pollutant concentrations, as well as differences in behavior and activities.

Although the OR estimates at lower levels seem negatively associated with the risk of AC in the exposure-response curves, their association is insignificant with a wide CI. We first suspect that the limited sample sizes in low-concentration exposure groups may introduce statistical uncertainty, resulting in wider CIs and potentially less precise estimates of the exposure-response relationship. Nonetheless, we cannot rule out the possibility of protective effects at low SO_2_ concentration levels, especially since previous studies suggest that low-level SO₂ exposure may contribute to vascular homeostasis by maintaining endothelial function and regulating hemodynamics [36]. Moreover, SO_2_ has been reported to have multiple protective mechanisms, such as antioxidant, anti-inflammatory, and anti-atherogenic effects [37, 38]. However, whether low levels of SO_2_ truly exert protective effects against AC still requires further study to be elucidated.

The possible mechanisms on SO_2_-induced conjunctiva damage may include inducing oxidative damage and inflammation in ocular, as well as irritating the eyes by lowering tear PH [37–39]. To further elucidate the link between SO_2_ exposure and AC, several biological pathways have been proposed. First, studies have shown that SO_2_-induced oxidative stress plays a crucial role in the pathogenesis of AC, leading to ocular surface damage and inflammation [38, 40, 41]. Second, as an acidic gas, SO_2_ has the potential to acidify tears, thereby reducing the pH of lacrimal and epithelial cells in the conjunctiva. This acidic environment can consequently disrupt the normal physiological function of the conjunctiva and promote the occurrence and development of AC [13]. Third, SO_2_ exhibits cytotoxic effects, including DNA damage and chromosome aberration. These cytotoxic events may disrupt the normal cellular processes in the conjunctiva and enhance the onset of AC [10, 38].

Our subgroup analyses revealed minimal heterogeneity by sex, age, or season, suggesting SO₂ effects on AC are broadly consistent across demographics. We found that the associations were significant in males but not in females, which aligns with previous studies [39]. This is possibly due to the fact that men were more likely to have higher outdoor activity levels than women, resulting in elevated risk of exposure. In terms of age, we observed slightly stronger effects in children under 6 or adults aged 18–60, whereas some studies highlight elders or adolescents as vulnerable groups. For example, a study conducted in Hangzhou reported more pronounced associations between SO_2_ and conjunctivitis visits in patients aged under 18 years or between 19 and 59 years [31]. Evidence from other cities of China also indicated that elders or children aged between 6 and 18 years may be more vulnerable [10, 33]. Notably, the above-mentioned studies were all conducted in one single city with varied demographic characteristic or SO_2_ levels, which may partially explain the inconsistence. In addition, our results suggested seasonal sensitivity in spring or autumn, which may be potentially due to increased outdoor activity in the two seasons, leading to higher exposure of ambient air pollutants.

This study presents several key strengths. First, to our knowledge, it represents the first multi-city study assessing the relationship between SO_2_ exposure and the risk of AC. This provides significant evidence of the harmful effects of SO_2_ on ocular surface. Second, the time-stratified case-crossover design enhance accuracy by effectively controlling for individual-level factors such as age, sex, socioeconomic status, and health conditions, as each case serves as its own control [19]. Additionally, this methodology inherently adjusts for long-term temporal trends and seasonal variations.

However, several limitations in our study warrant consideration. First, we utilized data on ambient pollutants and weather conditions from city-level monitoring stations as proxies for individual exposure due to the lack of direct measurements. While the limited number of stations may introduce exposure misclassification, this approach proven to be useful in many previous ecological studies [42, 43]. Additionally, this type of misclassification was assumed to be random and non-differential, thus likely biasing our effect estimates toward the null and making our reported associations more conservative [44, 45]. Second, the absence of precise AC onset times and locations for each patient could also introduce exposure measurement bias. Importantly, such biases typically produce nondifferential misclassification, either attenuating or maintaining the observed associations rather than creating spurious ones [46]. Third, it is possible that unmeasured or residual confounding factors may vary between case and control periods, which might influence the observed associations.

## Conclusion

This multi-city case-crossover study demonstrates that elevated ambient SO_2_ significantly increases the AC risk. Notably, the relationships remain consistent across sex, age and seasonal subgroups. In addition, the relationships persist even after adjusting for several major co-pollutants, reinforcing the independent role of SO_2_. This study highlights reducing SO_2_ exposure may be of great value in decreasing the risk of SO_2_ in AC pathogenesis. These findings underscore the potential benefits of reducing SO_2_ exposure in mitigating AC risks. While this study provides valuable epidemiological evidence, further research is needed to validate these results in diverse populations and elucidate the precise biological mechanisms linking SO_2_ exposure to ocular allergic response.

## Supplementary Information

Supplementary Material 1

## Data Availability

Data can be obtained from the corresponding author upon a reasonable request.

## Abbreviations

SO_2_: Sulfur dioxide
AC: Allergic conjunctivitis
ICD: The International Classification of Diseases
OR: Odds ratio
CI: Confidence interval
SD: Standard deviation
CO: Carbon monoxide
NO_2_: Nitrogen dioxide
PM_2.5_: Fine particulate matter
O_3_: Ozone
