# Supplementary material for: Ambient sulfur dioxide and daily outpatient visits for allergic conjunctivitis: a multi-city time-stratified case-crossover study in China
Source: BMC Public Health. 2025 Nov 22;26:390. doi: 10.1186/s12889-025-25700-x (PMC12853758; doi:10.1186/s12889-025-25700-x)
Supplement: Supplementary file 1 — Supplementary Material 1 [file 12889_2025_25700_MOESM1_ESM.pdf]

Contents

Figure S1. Map of the included cities with monitoring station points.

Figure S2. Time-series plot of the daily outpatient visit numbers for allergic conjunctivitis and the concentrations of ambient air pollutants in Guangzhou during the study period.

Figure S3. Time-series plot of the daily outpatient visit numbers for allergic conjunctivitis and the concentrations of ambient air pollutants in Huizhou during the study period.

Figure S4. Time-series plot of the daily outpatient visit numbers for allergic conjunctivitis and the concentrations of ambient air pollutants in Suzhou during the study period.

Figure S5. Time-series plot of the daily outpatient visit numbers for allergic conjunctivitis and the concentrations of ambient air pollutants in Wuxi during the study period.

Figure S6. Time-series plot of the daily outpatient visit numbers for allergic conjunctivitis and the concentrations of ambient air pollutants in Jinan during the study period.

Figure S7. Spearman correlation coefficients between air pollutants and meteorological factors in five cities during the study period.

Table S1. Summary statistics of daily air pollution and meteorological factors in five cities during the study period.

Table S2. The lag associations between each standard deviation increases in sulfur dioxide concentrations and outpatient visits for allergic conjunctivitis.

Table S3. The odds ratio of outpatient visits for allergic conjunctivitis with each standard deviation increases in the 8-day moving average concentrations of sulfur dioxide stratified by sex, age and season in five cities.

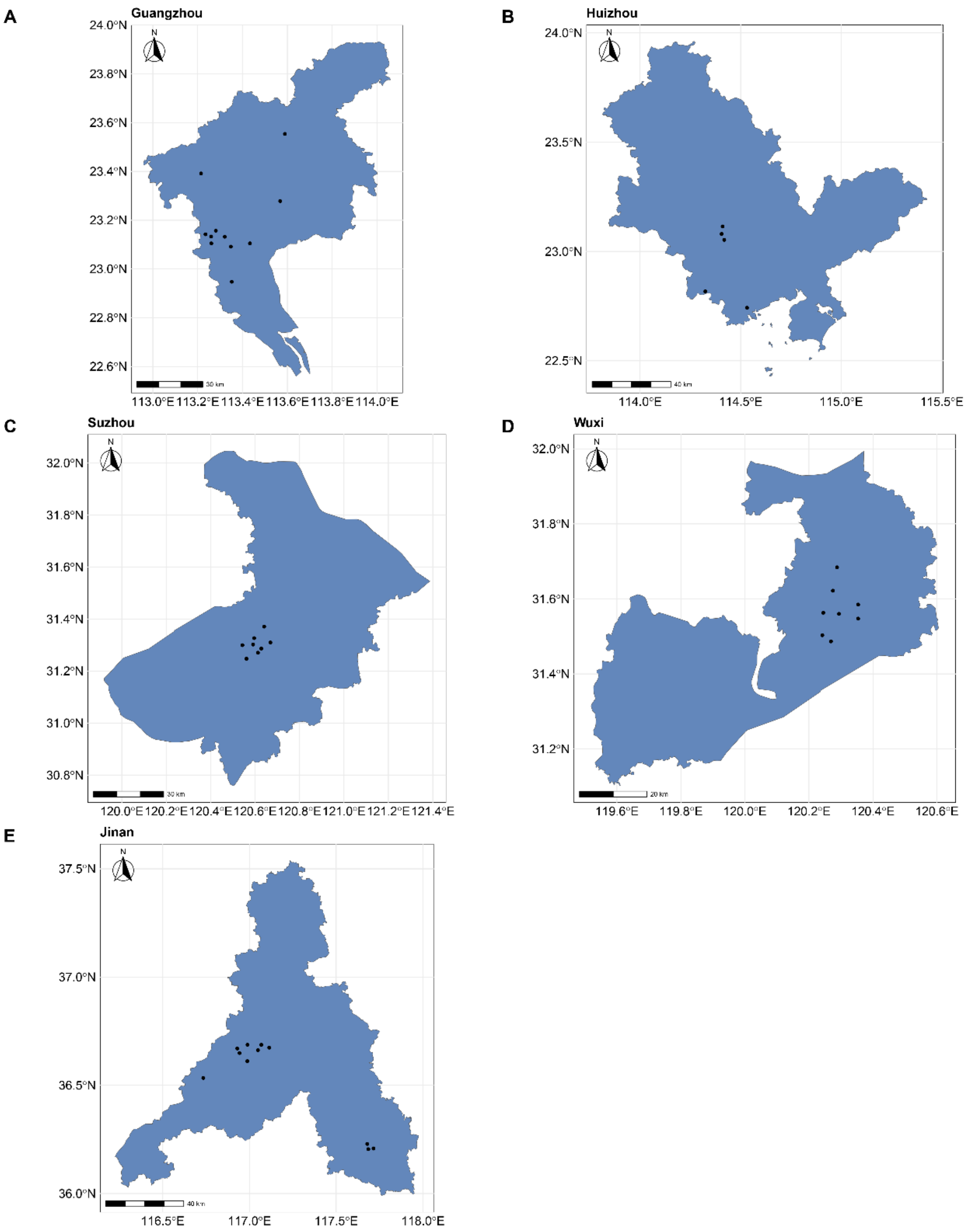

Figure S1. Map of the included cities with monitoring station points. The black dots indicating the location of monitoring stations.

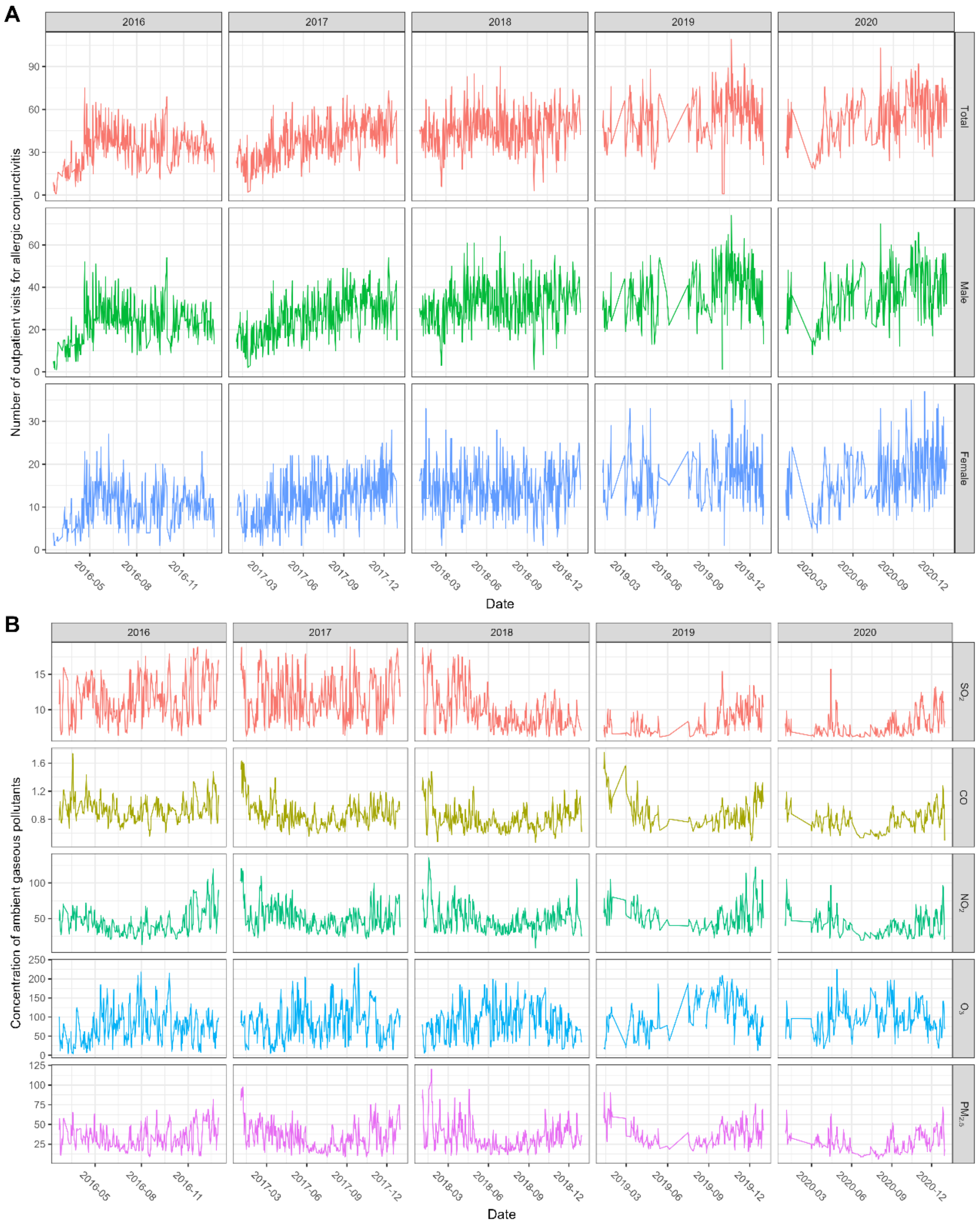

Figure S2. Time-series plot of the daily outpatient visit numbers for allergic conjunctivitis and the concentrations of ambient air pollutants in Guangzhou during the study period. (A) Daily outpatient visit numbers; (B) The concentrations of ambient air pollutants. The unit of measurement for the daily mean concentrations of SO_2, NO_2, $PM_{2.5}$ and the 8-h maximum mean level of O_3 were micrograms per cubic meter (μg/m^3), and for the daily mean level of CO was milligram per cubic meter (mg/m^3). Abbreviations: SO_2, sulfur dioxide; CO, carbon monoxide; NO_2, nitrogen dioxide; O_3, ozone; $PM_{2.5}$, fine particulate matter.

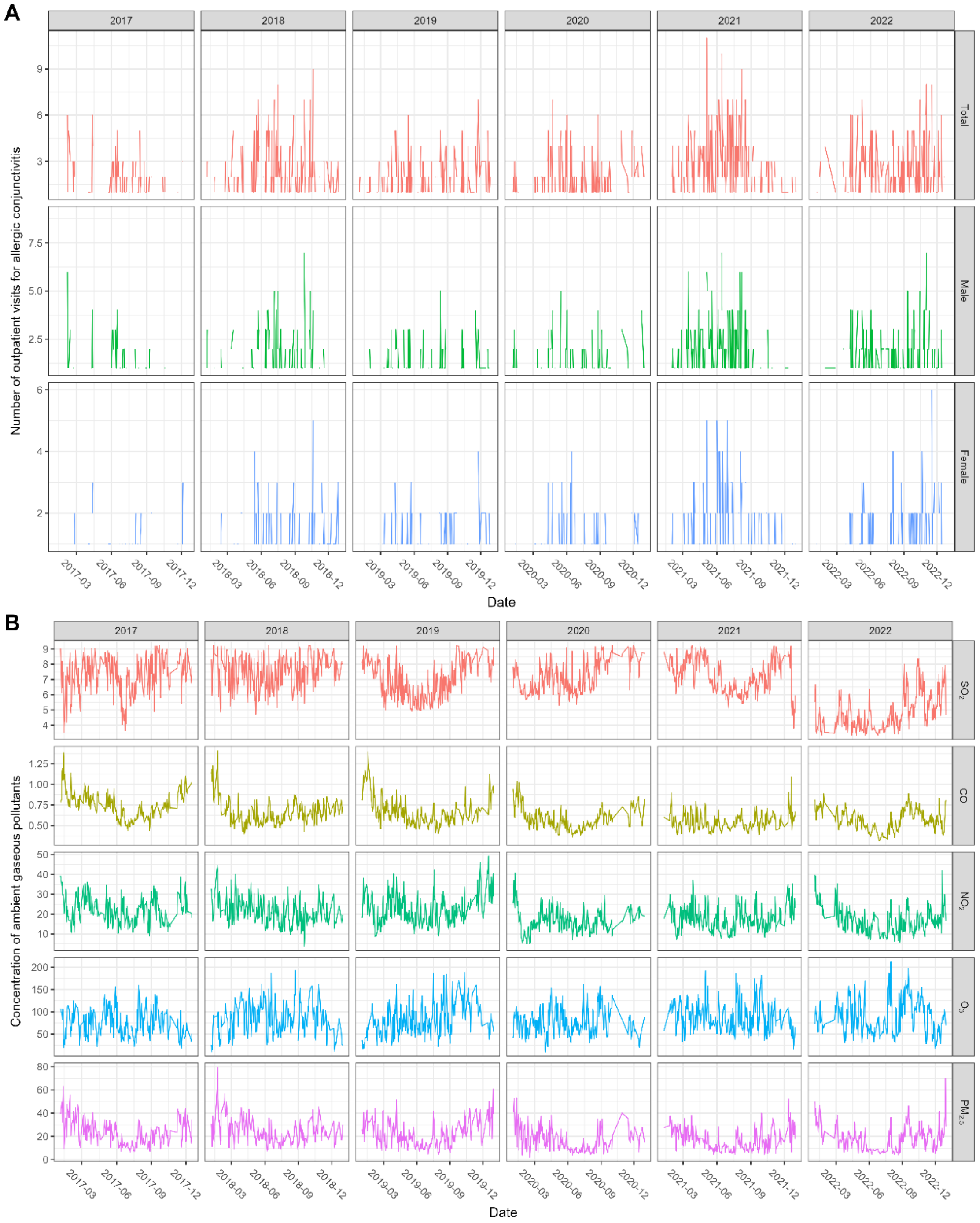

Figure S3. Time-series plot of the daily outpatient visit numbers for allergic conjunctivitis and the concentrations of ambient air pollutants in Huizhou during the study period. (A) Daily outpatient visit numbers; (B) The concentrations of ambient air pollutants. The unit of measurement for the daily mean concentrations of SO_2, NO_2, $PM_{2.5}$ and the 8-h maximum mean level of O_3 were micrograms per cubic meter (μg/m^3), and for the daily mean level of CO was milligram per cubic meter (mg/m^3). Abbreviations: SO_2, sulfur dioxide; CO, carbon monoxide; NO_2, nitrogen dioxide; O_3, ozone; $PM_{2.5}$, fine particulate matter.

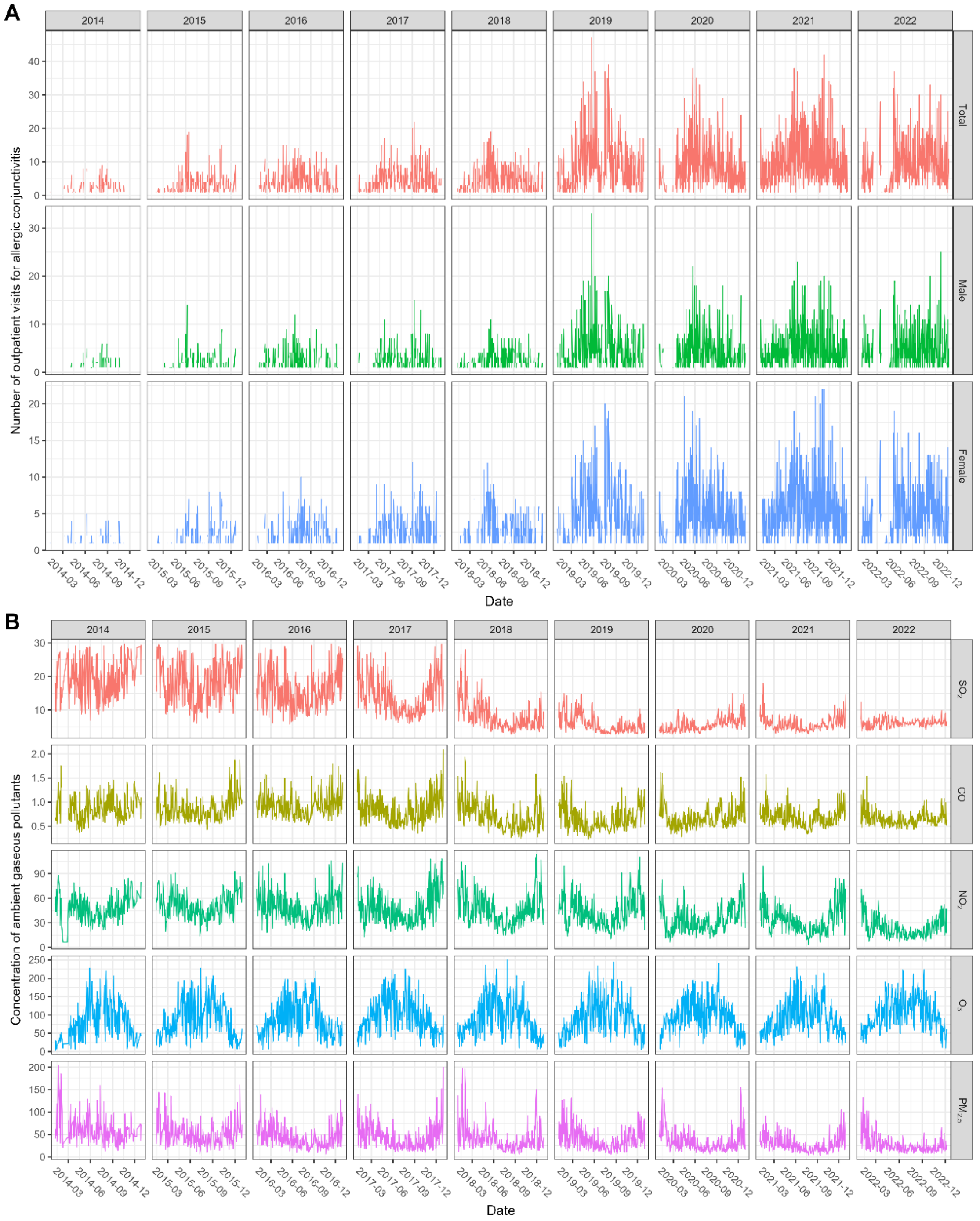

Figure S4. Time-series plot of the daily outpatient visit numbers for allergic conjunctivitis and the concentrations of ambient air pollutants in Suzhou during the study period. (A) Daily outpatient visit numbers; (B) The concentrations of ambient air pollutants. The unit of measurement for the daily mean concentrations of SO_2, NO_2, $PM_{2.5}$ and the 8-h maximum mean level of O_3 were micrograms per cubic meter (μg/m^3), and for the daily mean level of CO was milligram per cubic meter (mg/m^3). Abbreviations: SO_2, sulfur dioxide; CO, carbon monoxide; NO_2, nitrogen dioxide; O_3, ozone; $PM_{2.5}$, fine particulate matter.

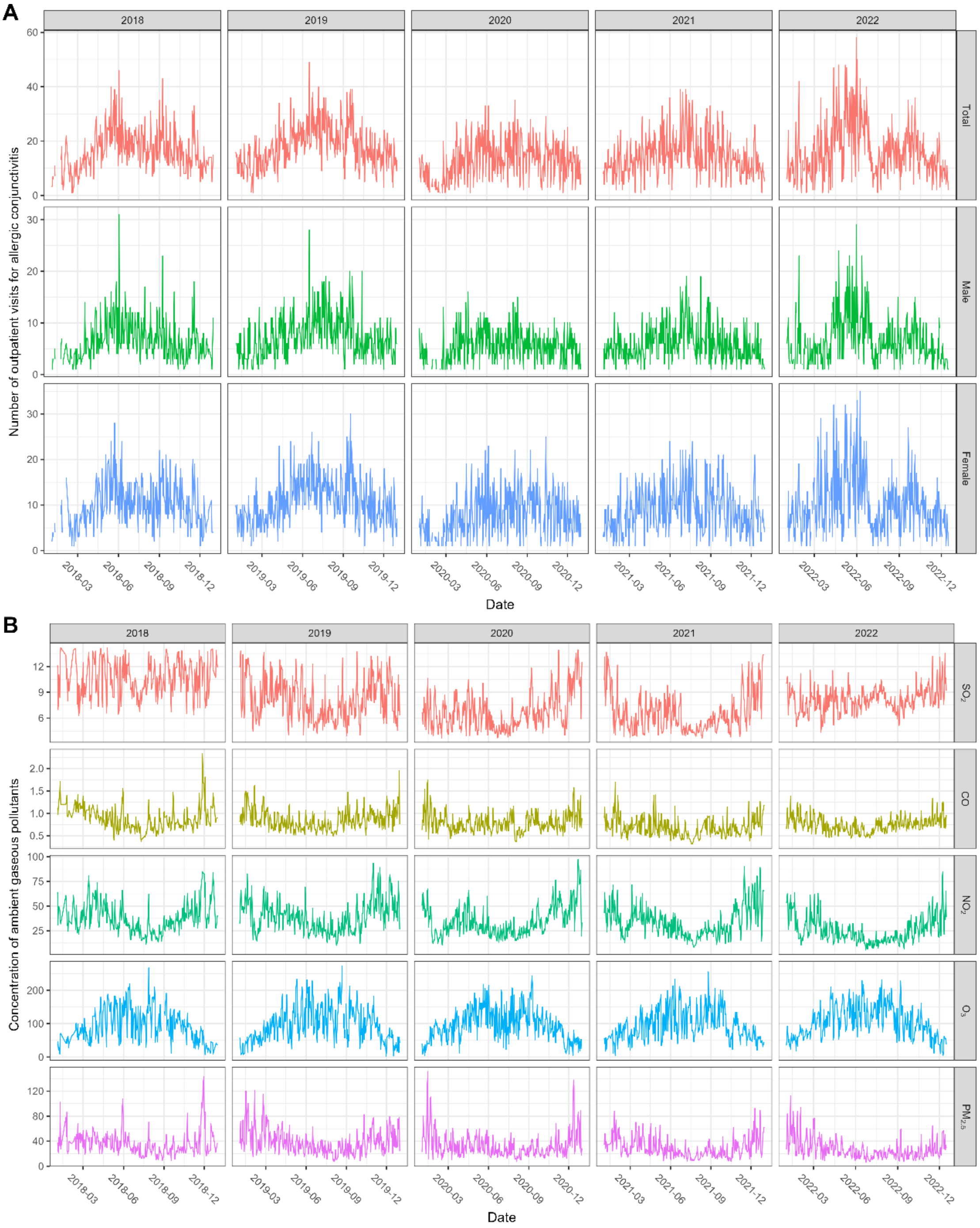

Figure S5. Time-series plot of the daily outpatient visit numbers for allergic conjunctivitis and the concentrations of ambient air pollutants in Wuxi during the study period. (A) Daily outpatient visit numbers; (B) The concentrations of ambient air pollutants. The unit of measurement for the daily mean concentrations of SO_2, NO_2, $PM_{2.5}$ and the 8-h maximum mean level of O_3 were micrograms per cubic meter (μg/m^3), and for the daily mean level of CO was milligram per cubic meter (mg/m^3). Abbreviations: SO_2, sulfur dioxide; CO, carbon monoxide; NO_2, nitrogen dioxide; O_3, ozone; $PM_{2.5}$, fine particulate matter.

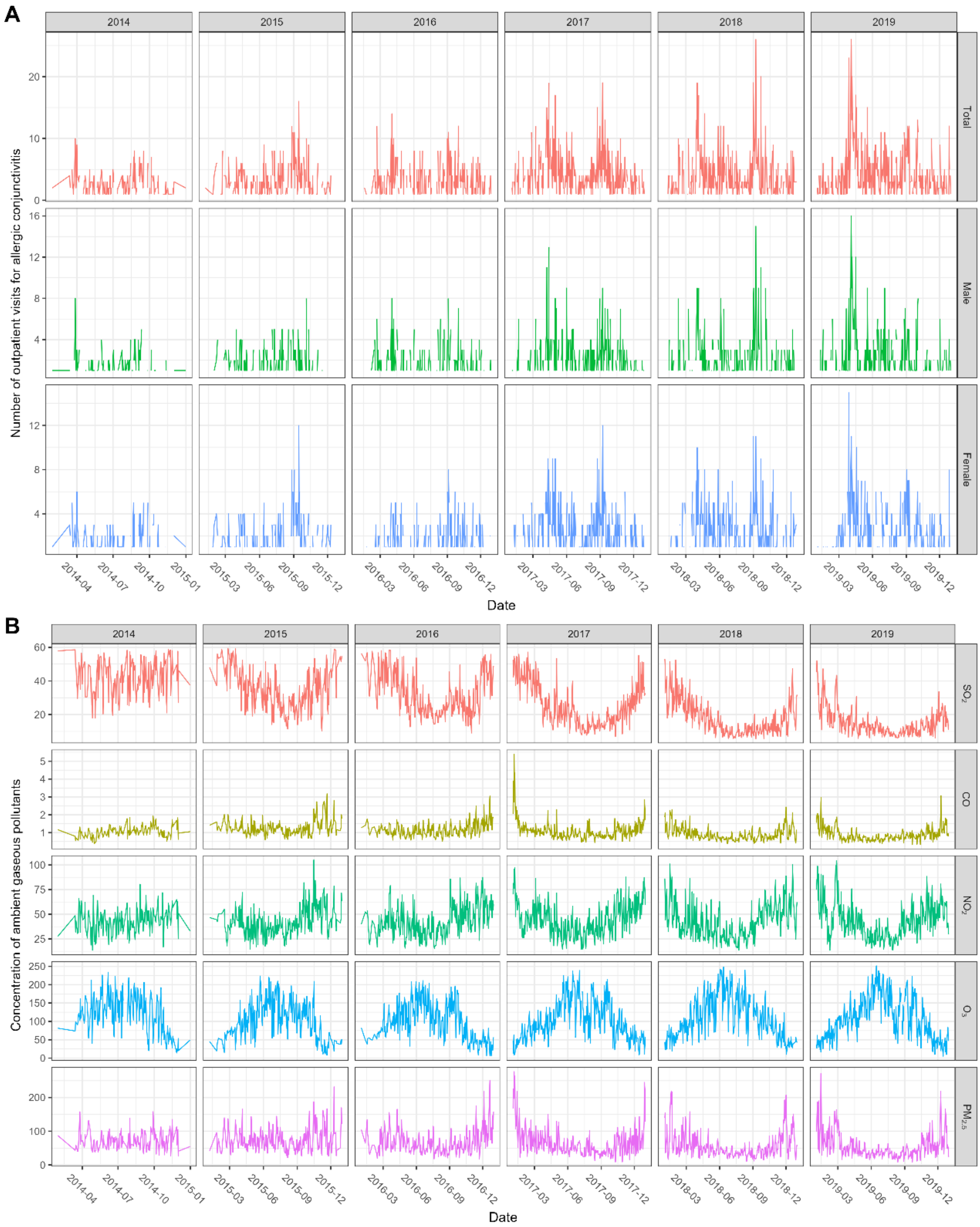

Figure S6. Time-series plot of the daily outpatient visit numbers for allergic conjunctivitis and the concentrations of ambient air pollutants in Jinan during the study period. (A) Daily outpatient visit numbers; (B) The concentrations of ambient air pollutants. The unit of measurement for the daily mean concentrations of SO_2, NO_2, $PM_{2.5}$ and the 8-h maximum mean level of O_3 were micrograms per cubic meter ($\mu g/m^3$), and for the daily mean level of CO was milligram per cubic meter (mg/m^3). Abbreviations: SO_2, sulfur dioxide; CO, carbon monoxide; NO_2, nitrogen dioxide; O_3, ozone; $PM_{2.5}$, fine particulate matter.

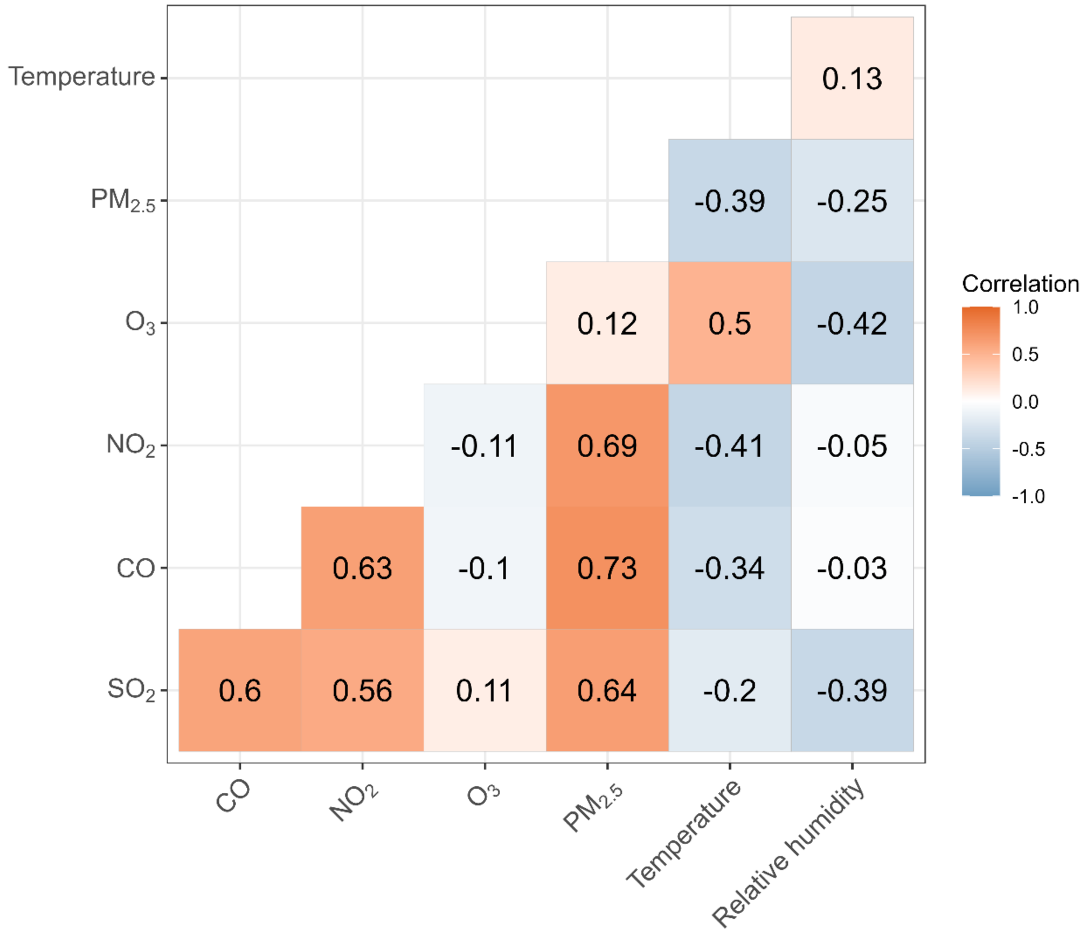

Figure S7. Spearman correlation coefficients between air pollutants and meteorological factors in five cities during the study period. Abbreviations: SO_2, sulfur dioxide; CO, carbon monoxide; NO_2, nitrogen dioxide; O_3, ozone; $PM_{2.5}$, fine particulate matter.

Table S1. Summary statistics of daily air pollution and meteorological factors in five cities during the study period.

Variables	Mean ± SD	Minimum	First quartile	Median	Third quartile	Maximum
**Guangzhou**						
SO_2 (μg/m^3)	10.1 ± 3.0	6.2	7.7	9.5	11.8	18.9
CO (mg/m^3)	0.9 ± 0.2	0.5	0.7	0.8	1.0	1.8
NO_2 (μg/m^3)	47.5 ± 17.8	8.6	34.8	44.0	56.8	135.2
O_3 (μg/m^3)	89.8 ± 45.7	4.1	54.8	87.8	119.4	240.2
$PM_{2.5}$ (μg/m^3)	33.1 ± 15.7	5.8	21.4	30.4	42.2	120.4
Temperature (°C)	22.9 ± 5.6	4.8	19.1	23.9	27.5	32.4
Relative humidity (%)	79.4 ± 11.4	27.7	73.3	81.3	87.3	99.0
**Huizhou**						
SO_2 (μg/m^3)	6.7 ± 1.4	3.3	5.8	6.7	7.8	9.3
CO (mg/m^3)	0.6 ± 0.2	0.3	0.5	0.6	0.7	1.4
NO_2 (μg/m^3)	19.1 ± 6.8	3.8	14.1	17.9	23.3	49.2
O_3 (μg/m^3)	82.3 ± 33.7	10.2	57.1	78.9	103.9	212.8
$PM_{2.5}$ (μg/m^3)	19.7 ± 10.3	2.0	11.6	18.1	25.6	79.6
Temperature (°C)	23.7 ± 6.2	3.9	19.3	25.4	28.8	33.5
Relative humidity (%)	74.9 ± 12	29.8	67.5	75.0	83.4	100.0
**Suzhou**						
SO_2 (μg/m^3)	10.4 ± 6.4	2.8	5.5	8.0	14.4	29.7
CO (mg/m^3)	0.7 ± 0.2	0.2	0.6	0.7	0.9	2.1
NO_2 (μg/m^3)	41 ± 18.3	3.2	27.3	38.7	52.0	113.0
O_3 (μg/m^3)	93.7 ± 46.3	4.1	58.5	88.0	126.1	249.9
$PM_{2.5}$ (μg/m^3)	39.5 ± 25	3.3	21.8	32.7	50.5	203.7
Temperature (°C)	18.2 ± 8.6	-6.3	11.0	19.0	25.1	35.3
Relative humidity (%)	76 ± 12.5	30.2	68.0	76.5	85.0	100.0
**Wuxi**						
SO_2 (μg/m^3)	7.9 ± 2.4	3.7	6.0	7.6	9.4	14.2
CO (mg/m^3)	0.8 ± 0.2	0.3	0.6	0.7	0.9	2.3
NO_2 (μg/m^3)	33.8 ± 15.6	5.4	22.0	31.5	43.1	97.2
O_3 (μg/m^3)	98.6 ± 48.8	2.9	60.2	91.1	132.9	274
$PM_{2.5}$ (μg/m^3)	32.5 ± 18.6	6.6	20.0	27.8	40.4	151.4
Temperature (°C)	18 ± 8.9	-6.6	10.4	18.4	25.6	35.0

Variables	Mean ± SD	Minimum	First quartile	Median	Third quartile	Maximum
Relative humidity (%)	73.6 ± 11.8	27.0	66.0	74.0	82.0	99.0
**Jinan**						
SO_2 (μg/m^3)	25.7 ± 13.6	5.7	14.3	23.0	35.5	59.4
CO (mg/m^3)	1.0 ± 0.4	0.3	0.8	1.0	1.2	5.4
NO_2 (μg/m^3)	43.2 ± 16.1	13.4	31.2	41.2	53.0	105.1
O_3 (μg/m^3)	107.6 ± 53.9	5.1	63.5	102.0	148.8	251.5
$PM_{2.5}$ (μg/m^3)	63.5 ± 35.8	8.6	38.8	54.5	80.0	276.8
Temperature (°C)	16.9 ± 10.1	-13.2	8.8	19.1	25.4	34.0
Relative humidity (%)	55.1 ± 19.2	14.3	39.9	53.7	68.8	100.0

Abbreviations: SO_2, sulfur dioxide; CO, carbon monoxide; NO_2, nitrogen dioxide; O_3, ozone; $PM_{2.5}$, fine particulate matter; SD, standard deviation.

Table S2. The lag associations between each standard deviation increases in sulfur dioxide concentrations and outpatient visits for allergic conjunctivitis.

Lag days	Guangzhou	Huizhou	Suzhou	Wuxi	Jinan	Overall
	OR (95% CI)	OR (95% CI)	OR (95% CI)	OR (95% CI)	OR (95% CI)	OR (95% CI)
Lag_0	1.017 (1.003, 1.031)	0.927 (0.851, 1.009)	1.015 (0.963, 1.070)	1.006 (0.982, 1.031)	0.971 (0.912, 1.033)	1.008 (0.993, 1.024)
Lag_1	1.018 (1.005, 1.032)	1.085 (1.001, 1.176)	1.045 (0.996, 1.097)	1.019 (0.999, 1.040)	1.064 (1.002, 1.130)	1.022 (1.012, 1.033)
Lag_2	1.008 (0.995, 1.020)	1.083 (1.003, 1.169)	1.045 (0.998, 1.094)	1.026 (1.006, 1.046)	1.091 (1.028, 1.159)	1.029 (1.010, 1.048)
Lag_3	1.019 (1.007, 1.032)	1.036 (0.961, 1.117)	1.014 (0.968, 1.063)	1.007 (0.987, 1.026)	0.984 (0.927, 1.046)	1.015 (1.005, 1.025)
Lag_4	1.000 (0.988, 1.012)	1.131 (1.050, 1.219)	1.041 (0.995, 1.089)	0.999 (0.980, 1.019)	0.958 (0.902, 1.018)	1.010 (0.989, 1.031)
Lag_5	1.003 (0.991, 1.016)	1.091 (1.015, 1.173)	1.012 (0.966, 1.059)	1.014 (0.995, 1.034)	0.959 (0.903, 1.019)	1.008 (0.994, 1.024)
Lag_6	1.001 (0.988, 1.013)	1.076 (0.999, 1.158)	1.080 (1.031, 1.131)	1.001 (0.981, 1.021)	0.990 (0.930, 1.053)	1.014 (0.995, 1.035)
Lag_7	1.007 (0.994, 1.020)	1.080 (0.999, 1.167)	1.021 (0.976, 1.068)	0.993 (0.974, 1.013)	1.008 (0.950, 1.069)	1.005 (0.995, 1.016)
Lag_8	0.997 (0.985, 1.010)	1.056 (0.979, 1.138)	1.012 (0.967, 1.059)	0.994 (0.975, 1.013)	1.016 (0.957, 1.080)	0.999 (0.989, 1.009)
Lag_9	0.994 (0.982, 1.006)	1.040 (0.966, 1.120)	1.027 (0.981, 1.075)	0.976 (0.957, 0.995)	0.985 (0.928, 1.046)	0.992 (0.979, 1.004)
Lag_{10}	0.980 (0.968, 0.992)	1.020 (0.947, 1.099)	1.002 (0.957, 1.050)	0.978 (0.960, 0.998)	0.960 (0.903, 1.020)	0.981 (0.971, 0.990)
Lag_{11}	0.992 (0.980, 1.004)	0.989 (0.917, 1.067)	1.019 (0.973, 1.068)	0.982 (0.963, 1.001)	0.943 (0.887, 1.004)	0.989 (0.980, 0.999)
Lag_{12}	0.997 (0.985, 1.010)	1.084 (1.007, 1.167)	0.997 (0.950, 1.046)	0.972 (0.953, 0.991)	0.966 (0.909, 1.027)	0.991 (0.973, 1.009)
Lag_{13}	0.998 (0.986, 1.011)	1.055 (0.980, 1.136)	0.986 (0.939, 1.036)	0.983 (0.964, 1.002)	0.944 (0.887, 1.005)	0.992 (0.979, 1.005)
Lag_{14}	0.993 (0.981, 1.006)	1.045 (0.969, 1.128)	1.018 (0.971, 1.067)	0.978 (0.959, 0.997)	0.966 (0.909, 1.027)	0.990 (0.979, 1.002)
Lag_{0-1}	1.024 (1.008, 1.039)	1.010 (0.918, 1.110)	1.044 (0.983, 1.108)	1.020 (0.993, 1.047)	1.024 (0.954, 1.100)	1.023 (1.010, 1.036)

Lag days	Guangzhou	Huizhou	Suzhou	Wuxi	Jinan	Overall
	OR (95% CI)	OR (95% CI)	OR (95% CI)	OR (95% CI)	OR (95% CI)	OR (95% CI)
Lag_{0-2}	1.023 (1.006, 1.040)	1.056 (0.954, 1.169)	1.063 (0.997, 1.134)	1.032 (1.003, 1.060)	1.075 (0.994, 1.164)	1.029 (1.015, 1.043)
Lag_{0-3}	1.031 (1.012, 1.049)	1.067 (0.957, 1.188)	1.062 (0.992, 1.138)	1.030 (1.001, 1.061)	1.059 (0.972, 1.153)	1.033 (1.019, 1.049)
Lag_{0-4}	1.028 (1.009, 1.048)	1.120 (1.001, 1.255)	1.078 (1.002, 1.161)	1.026 (0.995, 1.058)	1.035 (0.944, 1.134)	1.032 (1.016, 1.048)
Lag_{0-5}	1.028 (1.007, 1.049)	1.150 (1.022, 1.293)	1.080 (0.999, 1.168)	1.031 (0.998, 1.064)	1.015 (0.920, 1.120)	1.033 (1.016, 1.050)
Lag_{0-6}	1.027 (1.005, 1.049)	1.171 (1.037, 1.324)	1.117 (1.027, 1.214)	1.029 (0.995, 1.065)	1.012 (0.912, 1.124)	1.040 (1.013, 1.067)
Lag_{0-7}	1.029 (1.006, 1.053)	1.195 (1.052, 1.358)	1.128 (1.032, 1.232)	1.025 (0.989, 1.063)	1.016 (0.909, 1.134)	1.043 (1.013, 1.075)
Lag_{0-8}	1.028 (1.003, 1.053)	1.211 (1.059, 1.385)	1.133 (1.031, 1.245)	1.022 (0.984, 1.062)	1.023 (0.909, 1.152)	1.045 (1.011, 1.079)
Lag_{0-9}	1.025 (0.999, 1.051)	1.222 (1.063, 1.404)	1.146 (1.038, 1.264)	1.012 (0.972, 1.054)	1.017 (0.897, 1.153)	1.044 (1.006, 1.083)
Lag_{0-10}	1.016 (0.990, 1.044)	1.225 (1.060, 1.415)	1.147 (1.034, 1.273)	1.002 (0.961, 1.046)	0.999 (0.875, 1.140)	1.038 (0.997, 1.080)
Lag_{0-11}	1.013 (0.985, 1.042)	1.217 (1.048, 1.414)	1.159 (1.039, 1.293)	0.994 (0.951, 1.040)	0.975 (0.848, 1.121)	1.033 (0.990, 1.079)
Lag_{0-12}	1.012 (0.983, 1.042)	1.246 (1.067, 1.455)	1.162 (1.036, 1.304)	0.981 (0.936, 1.029)	0.959 (0.828, 1.111)	1.033 (0.983, 1.085)
Lag_{0-13}	1.011 (0.981, 1.043)	1.263 (1.077, 1.481)	1.161 (1.028, 1.311)	0.974 (0.927, 1.023)	0.926 (0.793, 1.080)	1.030 (0.976, 1.086)
Lag_{0-14}	1.008 (0.976, 1.041)	1.280 (1.085, 1.509)	1.177 (1.036, 1.337)	0.963 (0.915, 1.014)	0.905 (0.770, 1.064)	1.029 (0.969, 1.092)

Abbreviations: OR, odds ratio; CI, confidence interval.

Table S3. The odds ratio of outpatient visits for allergic conjunctivitis with each standard deviation increases in the 8-day moving average concentrations of sulfur dioxide stratified by sex, age and season in five cities.

Subgroups [a]	Guangzhou	Huizhou	Suzhou	Wuxi	Jinan
	OR (95% CI)	OR (95% CI)	OR (95% CI)	OR (95% CI)	OR (95% CI)
Sex					
Male	1.044 (1.014, 1.074)	1.324 (1.108, 1.582)	1.144 (1.001, 1.307)	1.024 (0.964, 1.087)	1.106 (0.930, 1.316)
Female	0.992 (0.950, 1.035)	1.073 (0.874, 1.317)	1.122 (0.982, 1.281)	1.021 (0.971, 1.072)	0.955 (0.813, 1.123)
Age, years					
<6	1.021 (0.990, 1.053)	1.337 (1.105, 1.616)	1.095 (0.928, 1.293)	1.133 (1.025, 1.253)	1.049 (0.781, 1.408)
≥6 & <18	1.034 (0.994, 1.075)	1.012 (0.791, 1.294)	1.160 (0.980, 1.375)	0.969 (0.898, 1.047)	1.133 (0.859, 1.494)
≥18 & <60	1.122 (0.914, 1.377)	1.212 (0.893, 1.645)	1.213 (1.027, 1.433)	1.039 (0.983, 1.099)	1.033 (0.882, 1.210)
≥60	1.158 (0.145, 9.261)	11.078 (0.035, 3500.142)	0.863 (0.565, 1.318)	0.937 (0.844, 1.041)	0.786 (0.534, 1.157)
Season					
Spring	0.989 (0.931, 1.051)	1.518 (1.154, 1.997)	1.148 (0.958, 1.375)	1.110 (1.020, 1.207)	1.107 (0.893, 1.372)
Summer	0.994 (0.933, 1.059)	1.294 (1.028, 1.629)	0.975 (0.804, 1.182)	1.007 (0.927, 1.094)	1.164 (0.893, 1.517)
Autumn	1.029 (0.988, 1.070)	1.020 (0.785, 1.325)	1.027 (0.817, 1.292)	1.065 (0.989, 1.146)	1.134 (0.844, 1.523)
Winter	1.111 (1.063, 1.161)	0.658 (0.460, 0.941)	1.188 (0.974, 1.449)	0.919 (0.848, 0.995)	0.685 (0.510, 0.920)

Abbreviations: OR, odds ratio; CI, confidence interval.
